# Impact of sensory modality and tempo in motor timing

**DOI:** 10.3389/fpsyg.2024.1419135

**Published:** 2024-08-09

**Authors:** Jaeuk Jeong, Soo Mi Nam, Hyejin Seo

**Affiliations:** ^1^Department of Physical Education, Seoul National University, Seoul, Republic of Korea; ^2^Division of Sports Science, Hanyang University, Ansan, Republic of Korea

**Keywords:** motor timing, sensorimotor synchronization, sensory modality, tempo change, circle drawing, virtual reality

## Abstract

**Background:**

Accurate motor timing requires the coordinated control of actions in response to external stimuli. Over the past few years, several studies have investigated the effect of sensory input on motor timing; however, the evidence remains conflicting. The purpose of this study was to examine the impact of sensory modality and tempo on the accuracy of timed movements and explore strategies for enhancing motor timing.

**Methods:**

Participants (*n* = 30) performed synchronization and adaptation circle drawing tasks in virtual reality. In Experiment 1, participants synchronized circle drawing with repeated stimuli based on sensory modalities (auditory, visual, tactile, audio-visual, audio-tactile, and visual-tactile) and tempos (20, 30, and 60 bpm). In Experiment 2, we examined timing adaptation in circle drawing tasks under conditions of unexpected tempo changes, whether increased or decreased.

**Results:**

A significant interaction effect between modality and tempo was observed in the comparison of timing accuracy. Tactile stimuli exhibited significantly higher timing accuracy at 60 bpm, whereas auditory stimuli demonstrated a peak accuracy at 30 bpm. The analysis revealed a significantly larger timing error when adapting to changes in the tempo-down condition compared with the tempo-up condition.

**Discussion:**

Through Experiment 1, we found that sensory modality impacts motor timing differently depending on the tempo, with tactile modality being effective at a faster tempo and auditory modality being beneficial at a moderate tempo. Additionally, Experiment 2 revealed that adapting to changes by correcting timing errors is more challenging with decreasing tempo than with increasing tempo. Our findings suggest that motor timing is intricately influenced by sensory modality and tempo variation. Therefore, to enhance the motor timing, a comprehensive understanding of these factors and their applications is imperative.

## 1 Introduction

Sensorimotor synchronization (SMS) is a time-related phenomenon that repeatedly coordinates motion with external cues. This phenomenon refers to the inherent rhythmicity and coordination of diverse human behaviors, including music, dance, walking, and sports motor skills. Proficiency in motor skills demands not only precise synchronization between external stimuli (e.g., sounds, light, and touch) and the internal rhythm and tempo of movements but also the ability to adapt to changes. This synchronization, known as perception-action coupling, involves interactive coordination between the sensory and motor areas of the nervous system. This process effectively stimulates sensorimotor circuits in the brain and promotes motor function (Oullier et al., 2005; Lakatos et al., 2019; Harry et al., 2023). For these reasons, SMS has demonstrated effectiveness in restoring lost motor function, making it a valuable intervention for conditions such as Parkinson’s disease, ADHD, and gait training (Arias and Cudeiro, 2008; Repp and Su, 2013; Rhea and Kuznetsov, 2017; Rosso et al., 2022).

Studies investigating the strength of perception-action coupling concerning sensory modalities have been conducted through research on motor timing (Elliott et al., 2010; Braun Janzen et al., 2014). We use “motor timing” to encompass the processes of sensorimotor synchronization and adaptation as it pertains to the coordination of motor output in response to external stimuli. Previous studies have shown that auditory stimuli are more effective in motor timing than other sensory modalities because of their higher temporal resolution and strong connection to the sensory and motor brain areas (Kolers and Brewster, 1985; Repp and Penel, 2004; Grahn et al., 2011; Varlet et al., 2012). However, the impact of sensory modalities on motor timing can vary depending on the individual characteristics or tasks (Repp and Su, 2013). In a study that evaluated the gait timing of individuals with Parkinson’s disease, it was observed that there was a synchronization improvement effect in the auditory modality, whereas this effect was not present in the visual modality (Arias and Cudeiro, 2008). In contrast, some researchers have shown that augmented visual information can assist in improving gait control timing in patients with vestibular dysfunction (Rhea and Kuznetsov, 2017).

Moreover, although multisensory inputs provide benefits in timing accuracy, negative consequences have been documented as a result of cognitive factors and sensory modality dominance. According to the optimal multisensory integration model, multiple sensory stimuli play a crucial role in timing control by enhancing the strong connections between perception and motor coordination through cross-reward interactions (Drugowitsch et al., 2014). In particular, multisensory integration has been reported to be more advantageous for SMS than a single sense under fast tempo conditions (Scott Kelso et al., 2001). However, combining three or more senses can lead to cognitive load, which has been reported to negatively impact timing performance (Ernst and Bülthoff, 2004; Elliott et al., 2010). Some studies have reported that when sensory inputs are combined, the dominance of a particular sensory modality can suppress the influence of other senses (Repp and Penel, 2004; Kato and Konishi, 2006). Additionally, it has been reported that even for the same combination of sensory modalities, timing control abilities vary depending on the task (Nieuwboer et al., 2007). The various outcomes of prior research on the impact of sensory modalities on motor timing and their combinations highlight the importance of investigating the optimal integration of sensory modalities to better comprehend and improve motor timing accuracy.

Maintaining coordinated motor timing not only involves accurate and consistent synchronization but also emphasizes the importance of rapid adaptation to changes. In sport area, varying the tempo to deceive opponents is a common strategy essential for both attackers and defenders. For instance, altering the dribbling tempo in basketball to execute a feint can deceive defenders. Similarly, in soccer and rugby, players adjust their tempo to disrupt the opponent’s rhythm or quickly adapt to changes made by attackers. To examine how individuals respond to such changes, motor timing research incorporates phase correction responses (PCR) as an adaptation indicator. PCR helps to investigate how individuals adapt to subsequent performances when unexpected tempo changes occur, providing valuable insights into their timing adaptability (Repp and Penel, 2002).

Previous research on SMS and motor timing has primarily focused on simple finger-tapping tasks, limiting their ecological validity and applicability to complex movements (Spencer and Ivry, 2005; Studenka and Zelaznik, 2011; Repp and Su, 2013). The constraint of these experimental tasks is that they measured the cognitive level of timing using a minimal range of motion with constraints on freedom. Therefore, to make them relevant to complex movements and sports-related actions, it is necessary to expand the range of motion and integrate movements with increased degrees of freedom. Circle drawing tasks are considered suitable as motor timing tasks, distinct from perceptual timing tasks like tapping, because they require the coordinated movement of multiple joints in the upper limb. For this reason, it has been used in numerous timing studies to assess the strength of perception-action coupling in upper limb motor skills (Zelaznik et al., 2000; Hiraga et al., 2005; Studenka and Zelaznik, 2011). Thus, to overcome the uncertainty regarding the effects of sensory modalities on motor timing and to enhance ecological validity and applicability, it is necessary to conduct a variety of sensory modalities in combination with expanded movements.

Conducting experiments to understand human psychological factors, such as the perception of timing, demands a high degree of sensitivity and rigorous control. Despite the need for precise control and a scientifically objective experimental design to distinguish between senses, previous research has been limited by the potential for sensory interplay in real-world environments (Braun Janzen et al., 2014). As an alternative solution, virtual reality (VR) offers advantages as a useful tool for immersive experiments, facilitating thorough and accurate evaluation of human motor behavior (Cohn et al., 2000). The use of VR, which allows for strict control over sensory precision, has the potential to be differentiated from previous research and ensure objectivity in this study.

In summary, despite extensive research suggesting that SMS is a promising approach for improving the timing of repetitive motor tasks, the optimal use of sensory modalities to enhance motor control remains unclear. Thus, the purpose of this study was to examine the accuracy and adaptability of motor timing with respect to perceptual information and to explore strategies for enhancing motor timing. To achieve this, in the first experiment, we aimed to compare how accurately individuals maintained their SMS based on sensory modality and tempo. In Experiment 1, we hypothesize that dual modality conditions (e.g., auditory–visual, auditory-tactile) will result in lower asynchrony values compared to single modality conditions (e.g., auditory, visual, tactile) across different tempos. However, single modality conditions are expected to show interaction effects of asynchrony depending on the tempo. In the second experiment, we assessed their ability to swiftly adapt to changes in tempo, either increasing or decreasing, using PCR. We hypothesize that timing adaptation in dynamically changing environments will encounter difficulties under slowing conditions, with interaction effects depending on the sensory modality. This demonstrates the strong coordination of complex biological phenomena, such as perception-action coupling.

## 2 Methods

### 2.1 Participants

Thirty healthy subjects (30 males, age: 26.3 ± 4.7 years) volunteered to participate in this study. All participants were confirmed to be right-handed using the Edinburgh Handedness Inventory (Oldfield, 1971). *A priori* power analysis with G*Power, version 3.1.9.7 (Faul et al., 2007) showed that a total of 30 participants would be required (*ρ* = 0.25, *α* = 0.05, 1−*β* = 0.95). We excluded participants who had difficulty performing the task because of their physical and/or neurological state. In addition, participants with prior experience with similar experiments were excluded. This study was approved by the Seoul National University Institutional Review Board (SNU IRB No. 1707/003-008) and all participants provided written informed consent prior to the experiment.

### 2.2 Apparatus and stimuli

The motor timing task was created using the Unity 3D program (Unity 3D, Unity Technologies, United States) and implemented using a VR device (HTC VIVE PRO-eye, HTC Corporation, Taiwan). Auditory stimuli were provided through the headset of the virtual reality equipment with a metronome. The head-mounted display (2,448 × 2,448 pixels resolution, 120 Hz frame refresh rate) provided a circle drawing task, and visual stimuli were presented as a flash on the target. Tactile stimuli were transmitted to participants through vibrations in the controller held in their right hand. We recorded the timing difference between the occurrence of the stimuli and the moment when the controller reached the red target in virtual reality.

**Figure 1:**
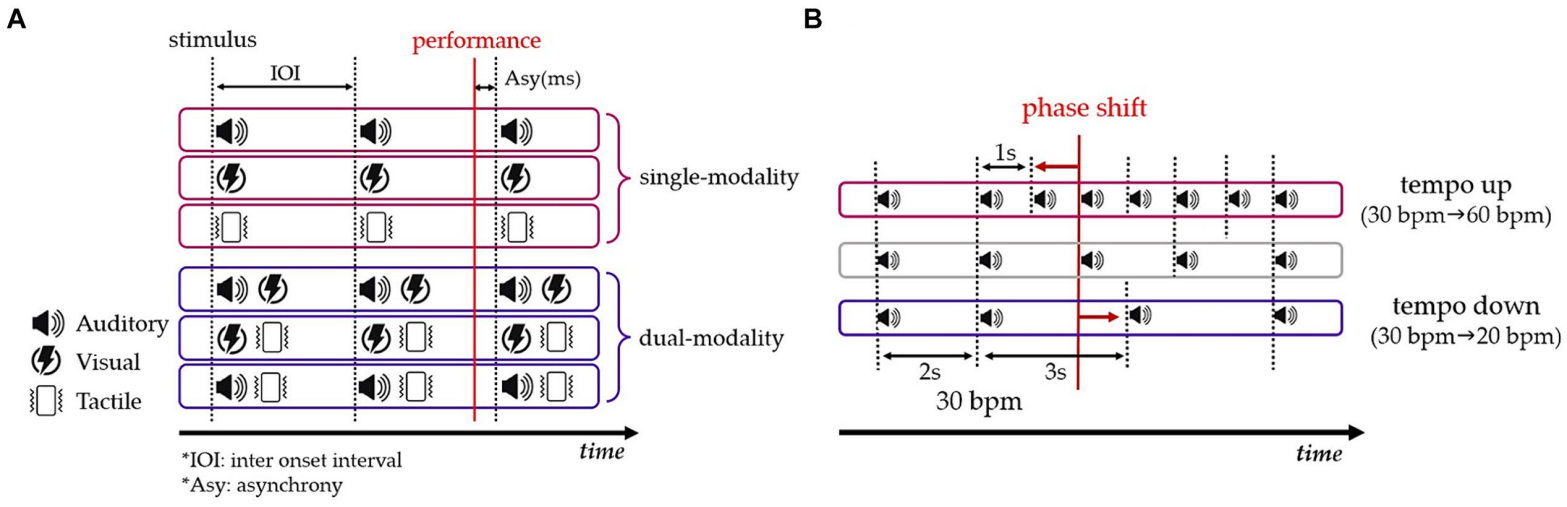
**(A)** Composition of sensory modality and timing error (Asy) in Experiment 1. **(B)** Set-up of tempo change conditions in Experiment 2. The tempo up condition exhibits a leftward phase shift (top), and the tempo down condition displays a rightward phase shift (bottom).

Experiment 1 consisted of six sensory modality conditions, including three single-modality and three dual-modality conditions. The single-modality condition involved continuous auditory signals (beeps), visual signals (flashes), and tactile signals (vibrations). The dual-modality condition combined different sensory modalities, including audio-visual, audio-tactile, and visual-tactile combinations (Figure 1A). The tempo was set based on an inter-onset interval (IOI) and was expressed in beats per minute (bpm) to signify the speed. Tempos were determined based on previous research. Our study required longer movement times due to larger trajectory sizes compared to a circle drawing timing study with an IOI of 500 ms (Studenka and Zelaznik, 2011). In cognitive timing research, IOIs typically range from 1,000 to 3,000 ms (Dione and Delevoye-Turrell, 2015; Roman et al., 2019). Therefore, considering both the movement size and the IOIs from previous studies, tempos were established as follows: 20 bpm (IOI = 3,000 ms), 30 bpm (IOI = 2000 ms), and 60 bpm (IOI = 1,000 ms). IOIs longer than 3,500 ms were restricted to prevent higher prediction bias (Bååth, 2016).

Experiment 2 was conducted under the same conditions as Experiment 1, but it included conditions in which the tempo changed in two directions (tempo up vs. tempo down). The tempo of the signals was intentionally varied in random timing, specifically in the 8th to 12th trials out of a total of 20 trials, following the methodology of a previous study (Studenka and Zelaznik, 2011). During these trials, the tempo was altered by either increasing or decreasing it. The tempo increase ranged from 30 to 60 bpm, whereas the tempo decrease ranged from 30 to 20 bpm (Figure 1B). This range of tempo variations allowed us to examine participants’ ability to adapt to and perceive changes in auditory stimuli throughout the experiment.

### 2.3 Task and procedure

**Figure 2:**
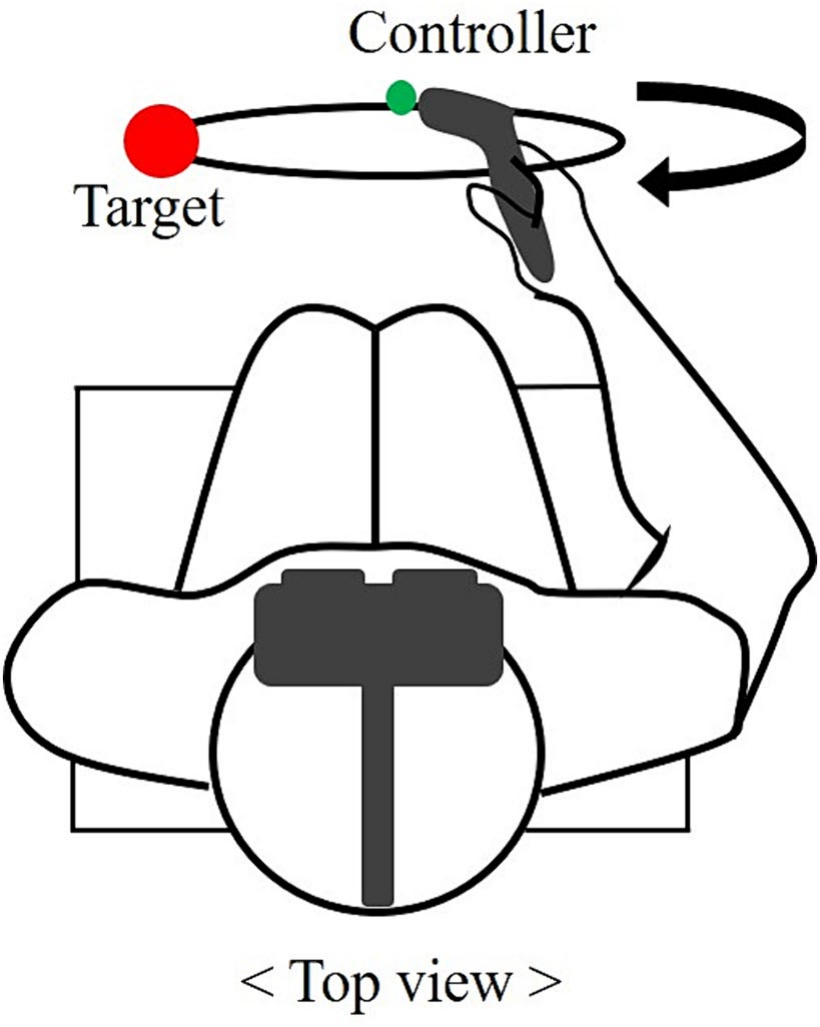
Top view of the circle drawing task. The red sphere represents the target and the endpoint of the controller is marked with a green dot. The motion of the clockwise rotation along the visible circle is depicted.

The experiment was conducted in an indoor laboratory equipped with a VR device. The participants wore virtual reality headsets and sat on chairs to maintain a comfortable state while preparing for the experiment. They were given five practice trials in the same experiment to adapt to virtual reality before the actual experiment. In the synchronization task (Experiment 1), participants synchronized their movements temporally and spatially with periodic stimuli under six conditions. The circle drawing traces a visually guided circle within the VR environment using a controller held in the right hand. The circular pathway was presented in the frontal plane, which was positioned 40 cm from the performer. To normalize the circle size, the diameter of the circle was matched to 50% of the participant’s arm length considering the range of participant motion. The target was a 10 cm diameter red sphere positioned at the 9 o’ clock position of the circle. The performers positioned the controller at the target and initiated the task after three preparatory signals, returning to the target point clockwise (Figure 2). During the task, they predicted the upcoming signal and aimed to synchronize it with precise timing.

The participants performed the tasks in an environment configured with six randomized stimulus conditions and three tempo conditions. Each of the 30 participants was assigned to one of six randomized orders, with five participants per order, ensuring that every participant experienced a different sequence. After five practice trials for each condition, designed to familiarize participants without allowing significant learning that could influence the sensitive auditory, tactile, and visual modalities, 20 repetitions were performed for each condition, with two blocks. To prevent the effects of fatigue and residual measurements, a five-minute rest period was provided between each condition following the protocol outlined by Zelaznik et al. (2000). In total, participants performed 720 circle drawing trials (6 modalities × 3 tempos × 20 trials × 2 blocks = 720), and the entire experiment took approximately 1 hour to complete. In the adaptation task (Experiment 2), the participants experienced randomized tempo changes during 20 circle drawing trials, specifically between the 8th and 12th trials. Upon detecting these changes, participants were instructed to adapt and proceed with the adaptation task. They performed 480 adaptation tasks (6 modalities × 2 shift directions × 20 trials × 2 blocks = 480), and the entire process took 30 min.

### 2.4 Data analysis

Unity 3D was programmed to extract the real-time 3D coordinates of the endpoint of the moving controller. The collected data from the endpoint were utilized to calculate the timing errors between the moment of signal generation and when the controller’s endpoint reached the target. In instances where participants did not reach the target, time error data could not be measured due to the absence of a target-reaching event. However, these instances constituted an average failure rate of approximately 2% of the total performances and were therefore excluded from the data analysis.

We defined asynchrony (Asy) as the average timing error between the target time and participants’ performance time. *A_i_* represents the actual performance time for the *i*-th sample, *T* denotes the target time, and *n* represents the total number of trials for each task (Equation 1).


(1) $$Asy=\overset{n}{\underset{i=1}{\sum }}\left|A_{i}-T\right|/n$$

The spatial error and variability were compared using the mean radial error (MRE), which measures the average deviation of the participant’s right-hand endpoint position (*performance_i_*) from the prescribed circular trajectory position (*target_i_*) on the frontal plane (Equation 2).


(2) $$MRE=\sqrt{\overset{n}{\underset{i=1}{\sum }}{\left(\mathrm{targe}t_{i}-\mathrm{performanc}e_{i}\right)}^{2}/n\;}$$

Phase correction response was calculated as the timing error observed in the next performance following a tempo-changing trial in an adaptation task. The variable *x_i_* represents the time of the *i*-th sample observed in the subsequent trial following the moment of phase shifting. The value *T* represents the target time, while *n* indicates the total number of conducted trials (Equation 3).


(3) $$PCR=\overset{n}{\underset{i=1}{\sum }}\left(x_{i}-T\right)/n$$

To compare motor timing based on sensory modalities and tempo in Experiment 1, we conducted a two-way repeated measures ANOVA with six modalities (visual, auditory, tactile, audio-visual, audio-tactile, and visual-tactile) and three tempos (20, 30, and 60 bpm). In Experiment 2, to examine the interaction between sensory modalities (six levels) and tempo changes (two levels: tempo up, tempo down), we conducted a two-way repeated measures ANOVA on the PCR variable. Post-hoc comparisons were conducted to investigate the main effects of sensory modalities, tempo, and phase shift. For interaction effects, we conducted multiple comparisons and applied the Bonferroni correction to regulate the significance level. Data refinement and analysis of temporal and spatial information were computed using the R package (ver. 4.1.2), and the significance level was set at 0.05.

## 3 Results

To understand the relationship between sensory information and motor timing, experiments were conducted in two stages. First, we investigated the impact of modality and tempo on sensorimotor synchronization. Second, we assessed the adaptive responses under changing conditions.

### 3.1 Experiment 1: the impact of sensory modality and tempo on sensorimotor synchronization

**Figure 3:**
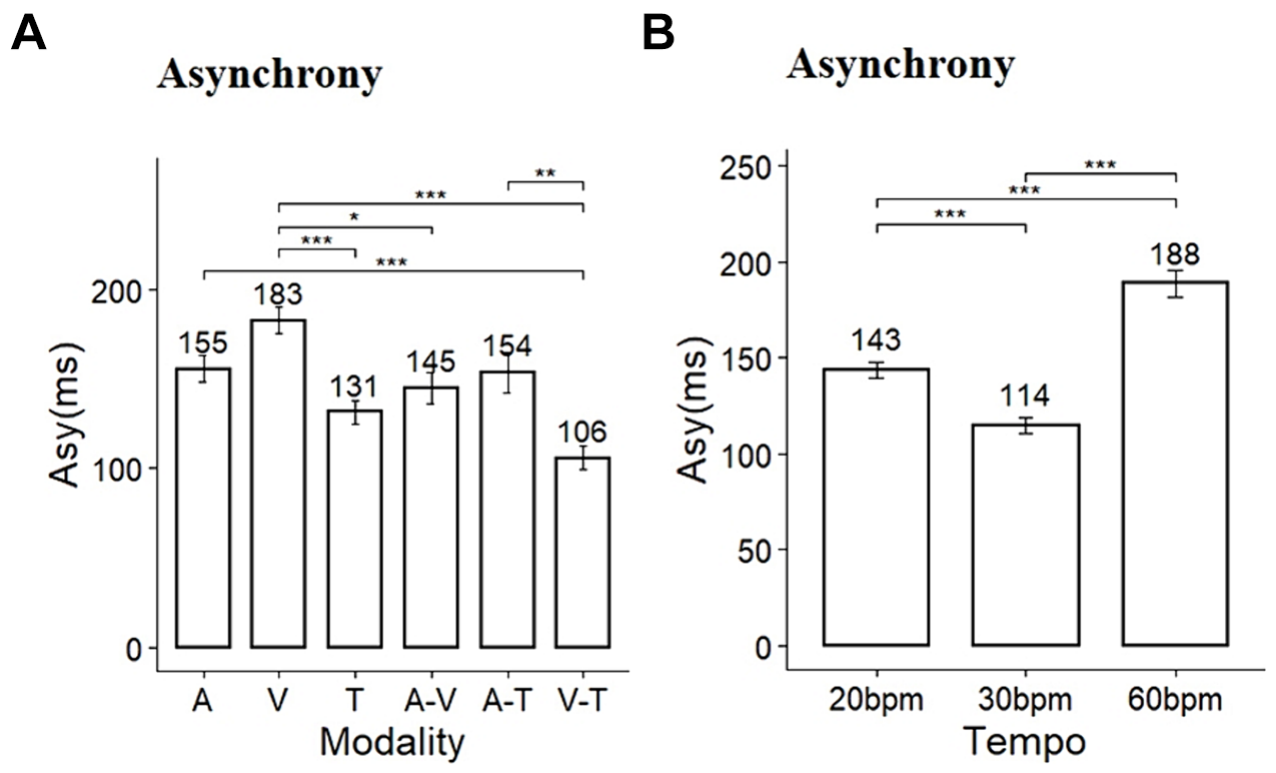
Comparison of asynchrony in **(A)** sensory modality and **(B)** tempo conditions (Asy, asynchrony; A, auditory; V, visual; T, tactile); ^*^ *p* < 0.05, ^**^ *p* < 0.01, ^***^ *p* < 0.001.

The results of the time error and statistical analysis for synchronization are presented in Table 1. Based on the time errors derived from the synchronization task, we evaluated the level of asynchrony. The sphericity assumption for the test of homogeneity of variance of the repeated factor was not violated, as evidenced by the Mauchly’s test of sphericity (*p* > 0.05). A two-way repeated measures ANOVA for asynchrony revealed significant main effects of sensory modality and tempo conditions, as well as a significant interaction between these factors (Modality: *F* (5, 145) = 24.16, *p* < 0.001, *η*^2^ = 0.22; Tempo: *F* (2, 58) = 28.958, *p* < 0.001, *η*^2^ = 0.52; Modality*Tempo: *F* (10, 290) = 12.6, *p* < 0.001, *η*^2^ = 0.22). Post-hoc comparisons for the main effects of modality showed significant differences in asynchrony across multiple pairs (Figure 3A). Tactile stimuli in single-modality and the combination of visual-tactile stimuli in dual-modality conditions resulted in the lowest asynchrony. And, except for the tactile condition, dual-modality conditions generally showed an advantage for timing accuracy. Post-hoc comparisons for the tempo revealed that asynchrony was significantly smaller at 30 bpm (mean = 114.9, SD = 56.3) than at 20 bpm (mean = 143.9, SD = 58.9, *p* < 0.001) and 60 bpm (mean = 188.5, SD = 95.4, *p* < 0.001) (Figure 3B).

**Table 1:** Analysis results of asynchrony (ms) by sensory modality and tempo.

Tempo	Modality	Mean (SD)	*F*	*p-*value
20 bpm	Auditory	139.1 (57.8)	1.77	0.12
	Visual	144.2 (43.7)		
	Tactile	160.0 (68.7)		
	Audio-Visual	135.9 (43.5)		
	Audio-Tactile	155.9 (83.6)		
	Visual-Tactile	117.3 (42.6)		
30 bpm	Auditory	108.4 (53.8)	4.78	<0.001^***^
	Visual	143.2 (55.7)		
	Tactile	120.0 (66.1)		
	Audio-Visual	97.8 (40.3)		
	Audio-Tactile	115.5 (52.8)		
	Visual-Tactile	77.6 (23.4)		
60 bpm	Auditory	219.0 (82.5)	18.16	<0.001^***^
	Visual	260.1 (70.2)		
	Tactile	113.8 (76.5)		
	Audio-Visual	200.6 (71.4)		
	Audio-Tactile	189.3 (106)		
	Visual-Tactile	122.3 (63.7)		

*** *p* < 0.001.

**Figure 4:**
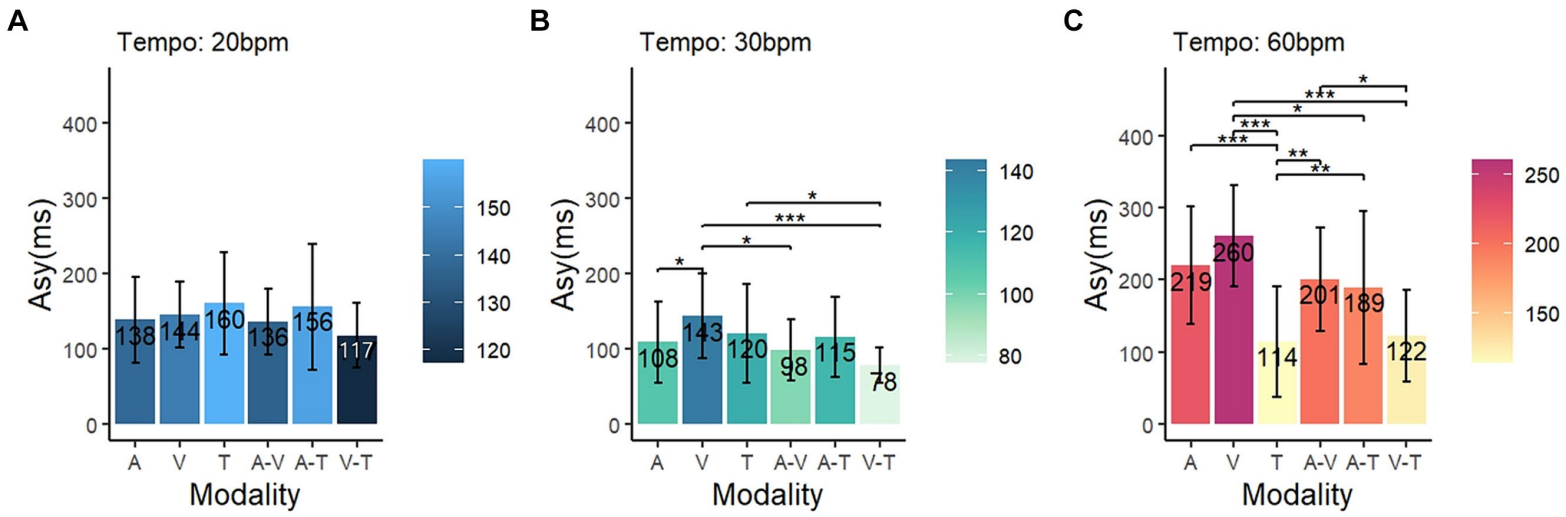
Comparison of asynchrony in each tempo condition. The blue, green, and red color schemes represent asynchrony at **(A)** 20 bpm, **(B)** 30 bpm, and **(C)** 60 bpm, respectively. The shading on the right side of each graph indicates the degree of asynchrony for the respective conditions (Asy, asynchrony; A, auditory; V, visual; T, tactile); ^*^ *p* < 0.05, ^**^ *p* < 0.01, ^***^ *p* < 0.001.

To compare the influence of sensory modalities based on specific tempo conditions, a simple main effect comparison analysis (multiple comparisons post-hoc test) was conducted. In the 20 bpm condition, the differences in asynchrony related to sensory modality were not significant (*p* = 0.12) (Figure 4A). Under the 30 bpm condition, the visual modality displayed significantly higher asynchrony compared to the auditory (*p* < 0.05), audio-visual (*p* < 0.05), and visual-tactile (*p* < 0.001) conditions (Figure 4B). Under the 60 bpm condition, the tactile modality exhibited significantly lower asynchrony than the other four modalities: audio-tactile (*p* < 0.01), audio-visual (*p* < 0.01), auditory (*p* < 0.001), and visual (*p* < 0.001) (Figure 4C). Conversely, asynchrony was higher in the visual modality than in the visual-tactile (*p* < 0.001) and audio-tactile (*p* < 0.05) modalities.

**Figure 5:**
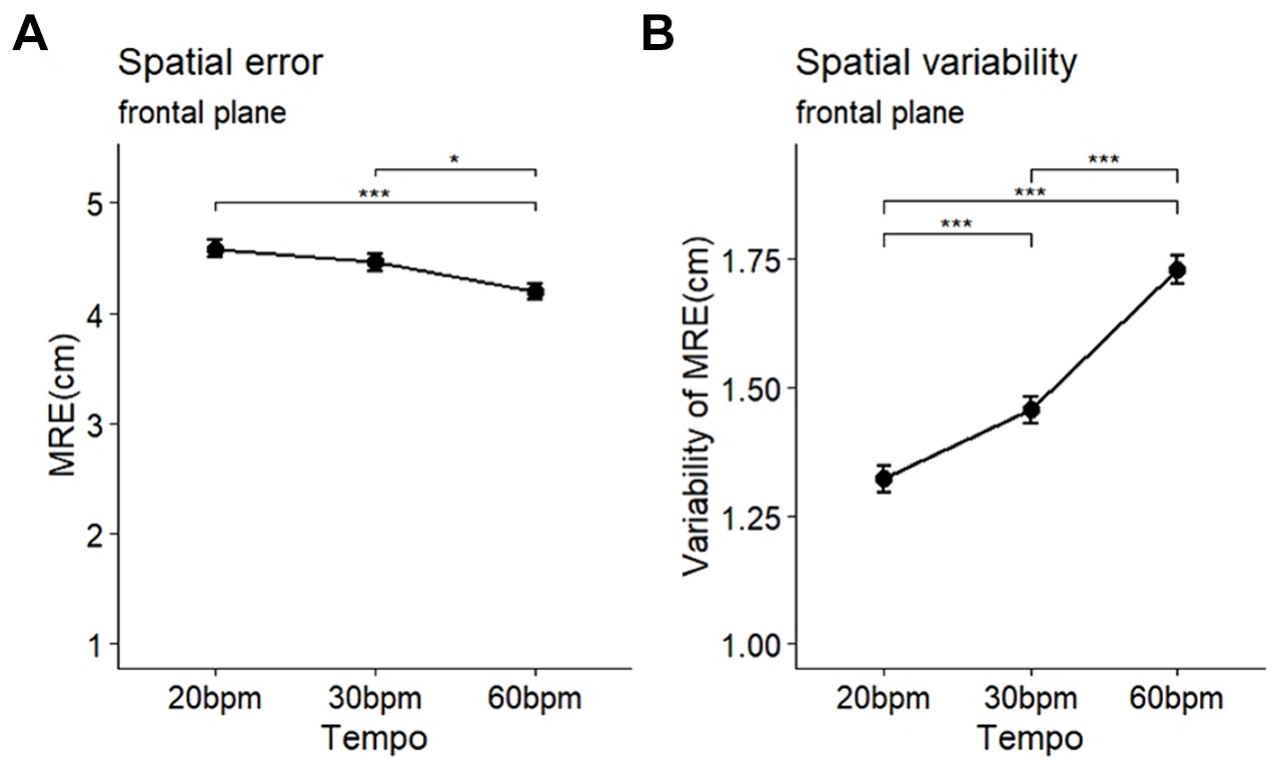
Comparison of the accuracy **(A)** and variability **(B)** of movement in spatial variables during a circle-drawing task on the frontal plane (MRE: mean radial error). *p < 0.05, ***p < 0.001.

The analysis of the spatial errors (MRE) during the circle-drawing task, according to tempo, revealed significant differences in both spatial errors and their variability (MRE: *F* (2, 58) = 7.10, *p* < 0.001, *η*^2^ = 0.025; MRE_variability_: *F* (2, 58) = 62.48, *p* < 0.001, *η*^2^ = 0.191) (Table 2). Post-hoc comparisons showed that the MRE at the 60 bpm (mean = 4.20, SD = 1.01) was significantly lower than at the 30 bpm (mean = 4.46, SD = 0.99, *p* < 0.05) and 20 bpm (mean = 4.59, SD = 0.99, *p* < 0.001) (Figure 5A). The variability of spatial errors also showed significant differences among all conditions (60 bpm > 30 bpm, *p* < 0.001; 60 bpm > 20 bpm (*p* < 0.001); 30 bpm > 20 bpm, *p* < 0.001) (Figure 5B).

**Table 2:** Analysis results of MRE (cm) and variability of MRE (cm) by tempo.

Variables	20 bpm	30 bpm	60 bpm	*F*	*p-*value
MRE (SD)	4.59 (0.99)	4.46 (0.99)	4.20 (1.01)	7.10	< 0.001^***^
MRE_variability_ (SD)	1.32 (0.35)	1.46 (0.35)	1.73 (0.36)	62.48	< 0.001^***^

*** *p* < 0.001.

### 3.2 Experiment 2: phase correction response to timing adaptation

The results of the time error and statistical analysis for the adaptation are presented in Table 3. Based on the time errors derived from the adaptation task, we evaluated the phase correction response. PCR values indicate the response to tempo change adjustments: positive values indicate a delayed reaction relative to the stimulus, whereas negative values suggest a tendency to anticipate and react ahead of the target timing. In Experiment 2, PCR values were negative for both tempo acceleration and deceleration conditions due to prediction. This prediction refers to the human motor system’s tendency to anticipate tempo changes and adjust in advance to compensate for the inherent delays in motor response, resulting in the performance reaching the target ahead of the stimulus timing.

**Table 3:** Analysis results of PCR (ms) by tempo change.

Phase shift	Modality	Mean (SD)	*F*	*p*-value
Tempo down (30 bpm ➔ 20 bpm)	Auditory	−241.6 (60.9)	8.417	<0.01^**^
	Visual	−235.2 (47.8)		
	Tactile	−315.6 (56.7)		
	Audio-Visual	−350.7 (58.1)		
	Audio-Tactile	−305.9 (59.2)		
	Visual-Tactile	−254.3 (52.5)		
	Average	−277.1 (55.5)		
Tempo up (30 bpm ➔ 60 bpm)	Auditory	−179.6 (11.2)		
	Visual	−48.4 (14.4)		
	Tactile	−210.4 (16.5)		
	Audio-Visual	−170.3 (14.8)		
	Audio-Tactile	−207.3 (10.8)		
	Visual-Tactile	−109.0 (25.6)		
	Average	−150.8 (16.4)		

** *p* < 0.01.

**Figure 6:**
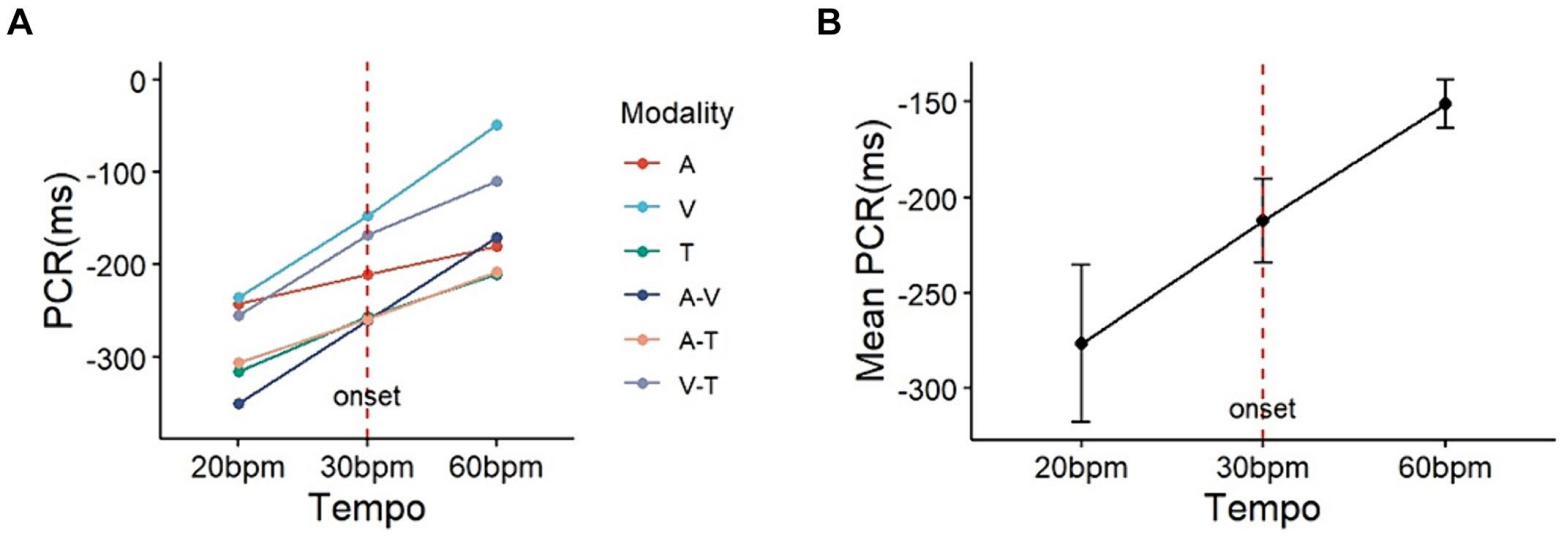
The plot on the left **(A)** represents the observed PCR for the six sensory modalities and **(B)** the right illustrates the average sensory modalities. Setting the baseline onset at 30bpm (red vertical dashed line), it can be observed that the change in PCR is greater under the condition of decreasing tempo to 20bpm compared to the condition of increasing tempo to 60bpm (PCR: phase correction response; A: auditory; V: visual; T: tactile).

Analysis of the PCR results (Table 3) indicated that the main effect of phase shift was significant (*F* (1, 29) = 8.417, *p* < 0.01, *η*2 = 0.023). The main effects of sensory modality and the interaction (phase shift*modality) were not significant (Modality: *F*(5, 145) = 0.960, *p* = 0.442, *η*2 = 0.013; Modality*Shift: *F* (5, 145) = 0.243, *p* = 0.943, *η*2 = 0.003). Post-hoc analysis for tempo change revealed that the absolute value of PCR under the tempo decrease condition (mean = 277.1, SD = 55.5) was significantly higher than under the tempo increase condition (mean = 150.8, SD = 16.4, *p* < 0.01) (Figure 6).

## 4 Discussion

This study examined the impact of perceptual information on the precision of timed movement. Particularly, in motor control, the ability to synchronize actions with external stimuli is referred to as motor timing. To explore the relationship between motor timing and sensory information, we conducted two distinct motor timing tasks.

### 4.1 Experiment 1: SMS depends on the sensory modality and tempo

The results of Experiment 1 confirmed that the participants exhibited significantly higher precision at 30 bpm (moderate tempo), whereas the highest level of time error was observed at 60 bpm (fast tempo). The speed-accuracy trade-off in motor tasks is well-documented, particularly in studies focusing on spatial accuracy (Fitts, 1954). However, previous research has suggested that faster tempos can enhance timing accuracy (Newell, 1980). Thus, our findings indicate that fast-paced movements negatively impact timing accuracy, which contrasts with the established understanding of the relationship between speed and timing accuracy. This discrepancy can be interpreted as follows: unlike previous research, the high degrees of freedom in circle drawing tasks introduce the difficulty of dual control. Participants must adhere to the provided circular trajectory while synchronizing their timing simultaneously. As the tempo increases, maintaining spatial accuracy becomes more challenging during faster movements, leading to a trade-off in timing accuracy.

Additionally, the findings suggest two potential reasons why synchronization errors were greater at 20 bpm compared to 30 bpm. First, at very slow tempos like 20 bpm, participants may struggle to maintain concentration and predict the timing of the next stimulus, which leads to higher timing errors. The increased interval between beats could disrupt their internal rhythm, making synchronization more challenging (Repp and Su, 2013; Harry et al., 2023). Second, at lower tempos, maintaining a consistent motion can cause variations in muscle tension and control, which in turn affects the precision of movements (De Graaf et al., 1991; Gorniak, 2019). These factors collectively contribute to the observed differences in synchronization errors between the two tempos.

A follow-up experiment can test how varying spatial control demands affect tempo management by having participants perform tasks of different spatial complexity while maintaining a consistent tempo. Motion capture would measure the impact of increased spatial complexity on tempo consistency, hypothesizing that higher complexity makes tempo maintenance more challenging. This builds on Newell’s findings, which showed effective motor control and timing in tasks with minimal spatial demands, providing insights into motor learning and coordination.

In analyzing the interaction effects of sensory modalities across the three tempo conditions, timing accuracy was highest for auditory at a moderate tempo, while tactile modality demonstrated greater accuracy at a fast tempo. These findings partially support previous research indicating that a high temporal resolution of auditory information is beneficial for timing (Repp and Penel, 2004; Elliott et al., 2010). However, it was observed to have limitations depending on the speed. In contrast to previous research, our study uncovered different results, indicating that tactile modality is more advantageous for timing at faster tempos. One explanation for this result is that proprioception, which is related to the sense of touch, may have a faster information-processing speed than visual and auditory modalities (Hillock-Dunn and Wallace, 2012). Therefore, proprioception could be advantageous for immediate response and synchronization in fast motor timing tasks. Additionally, under the slow tempo condition of 20 bpm, there were no significant differences in the timing results based on sensory modalities. This suggests that the distinct impact of a specific sensory modality is evident beyond a certain speed threshold.

Investigations comparing single and dual-modality conditions show that dual modalities (A-V, A-T, V-T) generally exhibit lower asynchrony values compared to single modalities (A, V, T), indicating better synchronization performance. When relying on a single modality, the brain depends heavily on the sensory information from that modality to predict and anticipate timing (Elliott et al., 2010; Zimmer and Macaluso, 2021). The brain’s ability to integrate multiple sources of information enhances the accuracy of timing predictions and the precision of motor outputs. This integration likely involves higher-order cortical processes where multisensory information is combined and processed to form a more accurate representation of temporal events (Keil and Senkowski, 2018). This suggests that even in tasks demanding high attention, the combination of two sensory modalities can improve timing accuracy through sensory integration. However, the exceptional finding that a single tactile modality was most advantageous for timing at a fast tempo implies a strong coupling between tactile sensation and motor timing.

### 4.2 Experiment 2: adaptive response to stimulus changes

Experiment 2 demonstrated that the magnitude of PCR values was larger under conditions where the phase decreased rather than increased at a consistent time. However, it was observed that the sensory modality had no impact on the speed variations of the adaptive timing task. The human motor system operates with inherent physical delays in response to stimuli, emphasizing the necessity of predicting timing synchronization. However, situations in which abrupt changes in unpredictable external events must be synchronized with actions require rapid correction responses. According to the sensory accumulation model (Aschersleben, 2002) and perceptual underestimation phenomenon (Wohlschläger and Koch, 2000), stimuli are typically perceived as occurring earlier than they are. Consequently, these early response adjustments yielded negative PCR results. Moreover, when transitioning to a slower tempo with more temporal variability, errors accumulate during the adjustment process, resulting in reduced timing accuracy.

In neuroscience, when dealing with time intervals of approximately 2 s or more, the influence of working memory becomes more prominent than the automatic control of shorter intervals (Koch et al., 2009). As performance time increases with a slower tempo, it can lead to a higher working memory load, similar to other cognitive processes. Rakitin et al. (1998) and Brown (2006) have shown that tasks requiring prolonged cognitive engagement time can increase working memory load. Furthermore, Buhusi and Meck (2005) discussed how working memory overload could impact timing predictions, leading to increased negative prediction errors. This increased reliance on working memory leads to a greater inclination for predictive responses to change, contributing to the noticeable negative phenomenon observed in timing adaptation. On the other hand, sensory modality has not been revealed to be a significant factor influencing timing adaptation. In research on motor timing and sensory perception, perception-action coupling is known to depend on sensory modality and plays a crucial role in timing control and adjustments (Repp and Keller, 2008). However, unlike sensorimotor synchronization in a stable tempo, timing control in changing environments does not show differences based on sensory modality. The fact that sensory modalities remain unaffected by timing accuracy in changing situations suggests that timing adaptability has unique characteristics compared to timing stability. Further discussion on predictive processes can underscore that while stable environments may rely heavily on specific sensory inputs for precise timing, adaptable environments require a more generalized predictive mechanism that is not modality-specific. This points to a more flexible and robust system of temporal processing that can handle variability and changes effectively.

## 5 General discussion

From an ecological perspective, perception and action are viewed as interdependent couplings (Gibson, 1979). The strength of perception-action coupling is associated with the ability to maintain stability or the tendency to restore to the original state. It is known that strong perception-action coupling is advantageous for maintaining stable movements, resisting perturbations, and making corrections to preserve stability. Therefore, examining the temporal aspects of SMS can provide evidence for inferring the stability of perception-action coupling.

Sensorimotor synchronization demands an internally stable tempo and rhythm while also requiring the ability to promptly correct errors. However, external information is diverse and human sensory processing operates in a complicated manner. Because of the complexity of the human system, it is challenging to comprehend how external information influences internal human processes. In this study, we aimed to overcome the limitations of previous research by comprehensively examining the integration of tempo and sensory information. Unlike Newell’s study (Newell, 1980), which utilized simple repetitive tasks such as key tapping, we sought to connect this with actual motor skills by expanding the degrees of freedom using the circle drawing task. Furthermore, by utilizing virtual reality, we ensured thorough control of the sensory input and immersion in the experiments, thus enhancing the validity of our research. Our findings suggest that motor timing intricacies involve a nuanced interplay among sensory modality, tempo, and adaptive responses to unexpected changes.

## Data Availability

The raw data supporting the conclusions of this article will be made available by the authors, without undue reservation.
